# Muscle matters: automated CT-based body composition analysis predicts survival in patients with head and neck cancer treated with immunotherapy

**DOI:** 10.3389/fonc.2026.1725892

**Published:** 2026-02-24

**Authors:** Frederic Jungbauer, Sonja Ludwig, Lena Huber, Annette Affolter, Anne Lammert, Nicole Rotter, Claudia Scherl, Elena Seiz, Farroch Vahidi Noghani, Stefan O. Schönberg, Johannes Haubold, Clara Arndt, Johannes M. Ludwig

**Affiliations:** 1 Department of Otorhinolaryngology, Head and Neck Surgery, University Medical Center Mannheim, Medical Faculty Mannheim of Heidelberg University, Mannheim, Germany; 2 Department of Radiology and Nuclear Medicine, University Medical Center Mannheim, Medical Faculty Mannheim of Heidelberg University, Mannheim, Germany; 3 Department of Diagnostic and Interventional Radiology and Neuroradiology, University Hospital Essen, University of Duisburg-Essen, Essen, Germany

**Keywords:** body composition analysis (BCA), computed tomography (CT) imaging, head and neck squamous cell carcinoma (HNSCC), immunotherapy, sarcopenia

## Abstract

**Purpose:**

The objective of this study was to evaluate the prognostic significance of CT-based body composition markers in patients with head and neck squamous cell cancer (HNSCC) treated with immunotherapy.

**Material and methods:**

Forty-five HNSCC patients (24.4% female, median age: 66 years) treated with Nivolumab or Pembrolizumab were retrospectively assessed. Automated body composition analysis was performed on thoracic CT scans. The analysis included skeletal muscle (SM), bone (B), visceral adipose tissue (VAT), and volumes of various adipose tissue compartments. Overall survival (OS) was estimated using the Kaplan-Meier method, and hazard ratios (HR) were calculated using the Cox proportional hazards model. In the multivariate analysis, the strongest body composition parameter was entered into the model.

**Results:**

The median OS was 8.13 months (95% CI: 4.8–21.9). Univariate analysis identified baseline high SM/B ratio, high (SM+VAT)/B ratio, albumin levels >3.4g/dl, low Eastern Cooperative Oncology Group (ECOG) performance status, body mass index ≥18.5 kg/m2, and male sex as significant prognostic factors for longer OS. In multivariate analysis, SM/B ratio (HR: 0.25, 95% CI: 0.1–0.64, p=0.004) and albumin ≤3.4 g/dl (HR: 0.31, 95% CI: 0.12–0.76, p=0.01) remained independent. Patients with both high SM/B and albumin survived the longest (median not reached) compared to either high SM/B or high albumin (9.6 months) vs. low SM/B and albumin (2.7 months). A decrease in SM/B of ≥ 8% after three months was associated with a lower median OS of 6.7 vs. 26.2 months, p=0.032.

**Conclusions:**

Automated CT-based body composition analysis, particularly the thoracic SM/B ratio and serum albumin level, provide valuable prognostic information on OS for HNSCC patients receiving immunotherapy and may guide clinical decision-making in this patient population.

## 1 Introduction

Head and neck squamous cell cancer (HNSCC) is a heterogeneous group of malignancies associated with significant morbidity and mortality. Traditionally, the management of these malignancies has involved multimodal approaches, including surgery, radiotherapy, and chemotherapy (1). The landscape of HNSCC treatment has undergone a paradigm shift in oncologic care due to the advent of immune checkpoint inhibitors, particularly programmed death-1 (PD-1) inhibitors such as Nivolumab and Pembrolizumab, resulting in improved survival outcomes in patients with recurrent and metastatic disease (2–5). Despite these therapeutic advances, patient outcomes remain variable, underscoring the need to identify reliable prognostic biomarkers that guide clinical decision-making and optimize treatment strategies.

The role of body composition and its impact on patients with cancer is increasingly acknowledged, with sarcopenia, defined by a gradual decline in skeletal muscle mass and function, as the key prognostic indicator in cancer care, influencing patients’ ability to tolerate treatment, their quality of life, and overall survival across multiple cancer types (6–8). Sarcopenia is notably common among individuals with HNSCC, with prevalence rates varying widely from 3.8% to 78.7%, depending on the evaluation methods and study populations (9). Its impact extends beyond muscle weakness, contributing to impaired mobility, heightened risk of falls and fractures, and acting as an independent risk factor for postoperative complications, chemotherapy-related toxicity, and treatment outcomes (6, 8, 10, 11).

Traditionally, body composition analysis using radiological methods has relied on manual segmentation of CT scans at the third cervical or third lumbar vertebrae, and rarely at the second thoracic vertebra, representing a time-consuming and inefficient approach for routine clinical practice (6, 12, 13). However, recent breakthroughs in automation, particularly those utilizing convolutional neural networks, have significantly transformed this field. These technologies now enable fast, consistent, and standardized measurements of muscle, fat, and bone across the entire CT scan, rather than limiting the assessments to a single slice (9, 14).

This study aimed to evaluate the prognostic significance of pretreatment CT-based body composition analysis of entire thoracic CT scans in patients with HNSCC treated with immunotherapy in terms of overall survival and time to progression.

## 2 Materials and methods

A total of 53 patients were administered immunotherapeutics at our institution between August 2018 and May 2023 for HNSCC treatment. Of these, 45 patients had thoracic CT images available for analysis. Clinical and laboratory data were extracted from the patient’s digital records. The final follow-up date for censoring was April 15, 2025. Ethical approval was obtained for this retrospective study, and the requirement for informed consent was waived (IRB Approval #: 2024-892).

### 2.1 Immunotherapy

Patients received a median of six (range: 1-47) treatments with either Nivolumab (Bristol-Myers Squibb, New York, New York, USA) (median: 6; range: 2-36) or Pembrolizumab (MSD, Kenilworth, New Jersey, USA) (median: 6; range: 1-47). Drugs were administered according to the manufacturer’s instructions. Treatment initiation and discontinuation were determined by an interdisciplinary tumor board. Tumor response and progression were defined based on a combination of clinical and radiological evaluations by an interdisciplinary tumor board. Immune-related adverse events (irAEs) were recorded according to the Common Terminology Criteria for Adverse Events v5.0.

### 2.2 Automated body composition analysis

**Figure 1:**
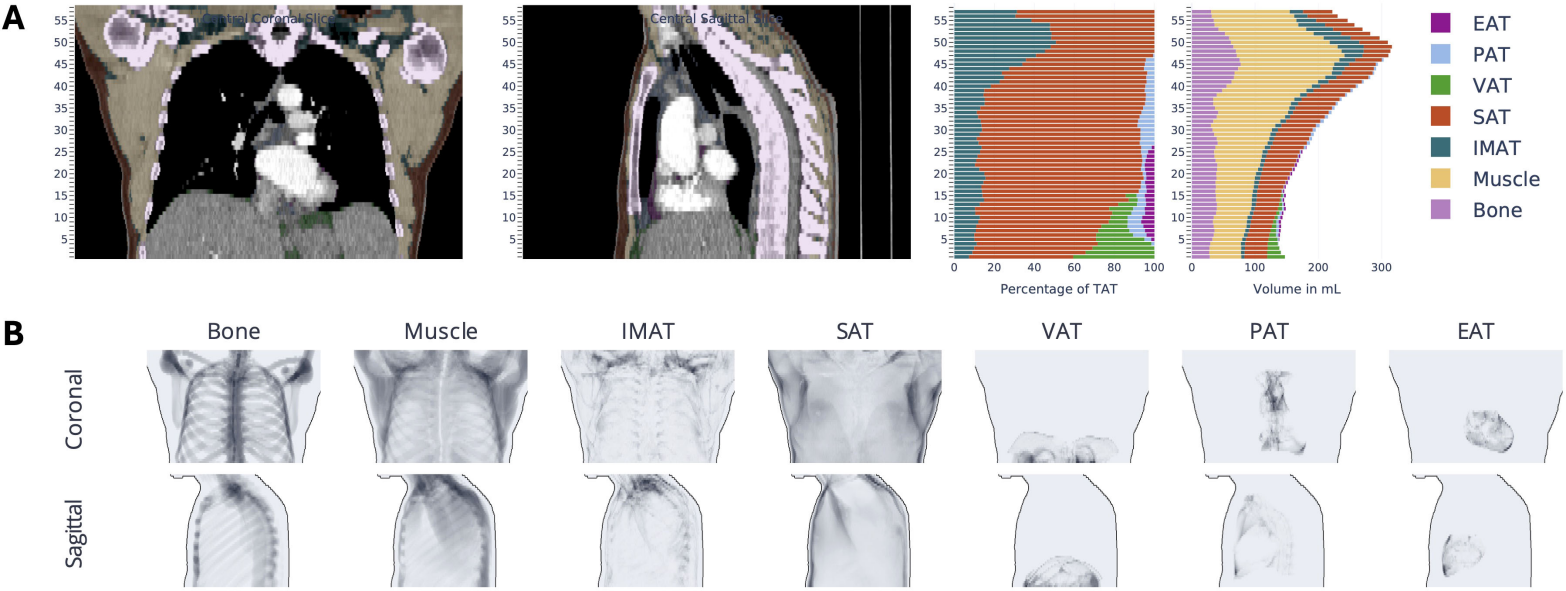
Visualization of CT-based body composition analysis.The following visual results are provided for the purpose of exemplifying a fully automated body composition analysis of thoracic CT scans. The results include the coloring of various tissues in the coronal and sagittal view, the general distribution map **(A)** and the presentation as a heat map **(B)** of total adipose tissue (TAT). Visceral adipose tissue (VAT), subcutaneous adipose tissue (SAT), epicardial adipose tissue (EAT), pericardial adipose tissue (PAT), intra- and intermuscular adipose tissue (IMAT), skeletal muscle (SM), and bone.

Automated body composition analysis was conducted using a fully automated algorithm for CT data interpretation, as described previously (14–17). The open-source software code can be retrieved at https://github.com/UMEssen/Body-and-Organ-Analysis. The applied version was v0.1.3. This approach utilizes a pretrained convolutional neural network to quantitatively assess the 3D volumes of various body tissues from standard thoracic CT images. To maintain uniformity regardless of the original slice thickness, the algorithm resampled all scans to 5 mm slices before analysis. The algorithm outputs several body composition metrics in the form of an average volume per CT slice, including total adipose tissue (TAT), visceral adipose tissue (VAT), subcutaneous adipose tissue (SAT), epicardial adipose tissue (EAT), pericardial adipose tissue (PAT), intra- and intermuscular adipose tissue (IMAT), skeletal muscle (SM), and bone (B) volumes (**Figure 1**). To account for variations in patient size and scanned regions, the analysis normalizes these parameters by automatically identifying the thoracic cavity, which includes part of the upper abdomen, and dividing the measured volumes by the number of 5 mm slices in this region, yielding mean values for each parameter. Furthermore, body composition parameters were standardized using bone volume measurements.

### 2.3 Statistics

The median overall survival (OS) and time to progression (TTP) were estimated using the Kaplan-Meier method, and the log-rank test was used to assess the significance of the differences. The area under the curve (AUC) was calculated for 3-month survival, and the Youden index was used to determine the cutoffs for all patients and sex-specific groups. For univariate (UVA) and multivariate (MVA) analyses, the Cox proportional hazards model was used to calculate hazard ratios (HR) and 95% confidence intervals (95% CI). Albumin and Body Mass Index (BMI, kg/m²) values were dichotomized at the lower end of the normal range. Contingency analysis was conducted using Pearson’s correlation coefficients. The two groups were compared using the Mann-Whitney U-test. Owing to the exploratory nature of this study, no alpha error correction was performed. Statistical significance was set at p < 0.05. Statistical analyses were performed using JMP 18.2 software (SAS Institute Inc., Cary, NC, USA).

## 3 Result

### 3.1 Baseline characteristics

A total of 45 patients (24.4% female) with a median age of 66 years (range: 44–85 years) were included in this study. The most common tumor site was the oropharynx (55.6%), followed by the hypopharynx (15.6%), larynx (11.1%), oral cavity (8.9%), and other locations, including carcinoma of unknown primary and sinus cancer (8.9%). The majority of patients (75.6%) presented with advanced-stage disease (stages III and IV). The baseline characteristics of all patients are shown in **Table 1**.

**Table 1:** Patients’ baseline characteristics.

Baseline characteristics	Patients
Gender	
• Male	75.6%
• Female	24.4%
Primary tumor location	
• Oropharynx	55.6%
• Hypopharynx	15.6%
• Larynx	11.1%
• Oral cavity	8.9%
• Other	8.9%
Tumor stage (UICC)	
• I	9.1%
• II	13.6%
• III	13.6%
• IV	63.6%
p16-Status	
• Positive	24.4%
Noxious agents	
• None	22.2%
• Smoking	31.1%
• Alcohol	8.9%
• Smoking and alcohol	37.8%
Previous treatments*	
• Surgery	16.3%
• Surgery + (C)RTx	30.6%
• CRTx	36.7%
• Palliative CTx/RTx	14.3%
• None	2%
Administered drug	
• Nivolumab	33.3%
• Pembrolizumab	66.7%
ECOG	
• 0	31.1%
• 1	42.2%
• 2	13.3%
• 3	6.7%
• 4	4.4%
• Unknown	2.2%

Baseline characteristics of the patients. *Four Patients received more than two prior therapies. ECOG, Eastern Cooperative Oncology Group; UICC, Union for International Cancer Control; (C)RTx (Chemo-) Radiotherapy; RTx, Radiotherapy; CTx, Chemotherapy.

### 3.2 Survival analysis

**Figure 2:**
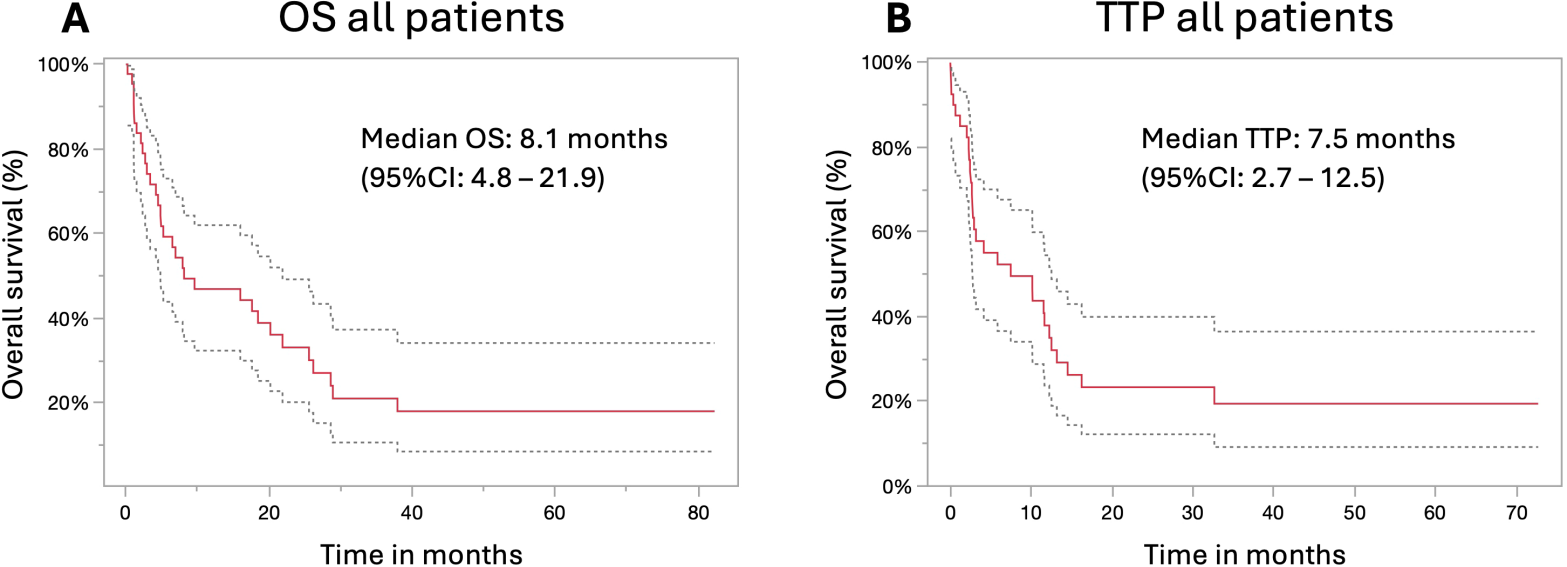
Overall survival and time to progression of the study cohort. Overall survival **(A)** and time to progression **(B)** with 95% confidence interval (95% CI; dotted line) of the study cohort following the first treatment with immunotherapy.

In thirty-two patients, death was recorded after a median time of 5.1 months (range: 0.2–38 months), while 13 patients were lost to follow-up after a median of 20.9 months (range: 0 – 82.3 months). The estimated median overall survival of all patients in this study was 8.13 months (95% CI: 4.8–21.9), with 1-month, 3-month, 6-month, 1-year, 2-year, and 3-year survival rates of 93.3%, 68.9%, 55.6%, 42.2%, 26.7%, and 17.8%, respectively (**Figure 2A**).

When evaluating body composition parameters as continuous variables, only the SM/B (p = 0.0053) and (SM + VAT)/B (p = 0.0005) ratios were significant in the univariate analysis of all patients. In the subanalysis, however, statistical significance was observed only in men but not in women (**Table 2**). Notably, the median values were significantly lower in females for the SM/B (1.44 vs. 1.69; p = 0.003) and (SM + VAT)/B (1.51 vs. 1.93; p = 0.001) ratios (see also **Supplementary Table 1** for all parameters). The AUC analysis revealed significance for the SM/B and (SM + VAT)/B ratios, with AUCs of 0.74 and 0.76, respectively (p ≤ 0.0069) for all patients. In the subanalysis, the AUC curves for both parameters were significant only in males, not in females. The complete data on AUC analysis of body composition parameters are presented in **Supplementary Table 2**. Thus, SM/B and (SM + VAT)/B were included in the Cox proportional hazards analysis with sex-specific cutoffs (**Table 3**).

**Table 2:** Overall survival univariate analysis of continuous body composition parameters.

Body composition	All patients		Male patients		Female patients	
	HR (95% CI)	P-value	HR (95% CI)	P-value	HR (95% CI)	P-value
SM/B	0.17 (0.05 – 0.59)	**0.0053**	0.21 (0.05 – 0.93)	**0.039**	0.25 (0.01 – 5.7)	0.37
TAT/B	0.93 (0.71 – 1.16)	0.5304	0.85 (0.59 – 1.13)	0.3	0.98 (0.48 – 1.84)	0.96
IMAT/B	0.90 (0.29 – 2.4)	0.84	0.8 (0.2 – 2.43)	0.72	0.12 (<0.001 – 15.7)	0.38
SAT/B	0.93 (0.61 – 0.62)	0.7	0.76 (0.4 – 1.2)	0.27	1.07 (0.42 – 2.5)	0.88
VAT/B	0.18 (0.017 – 1.07)	0.06	0.24 (0.018 – 1.59)	0.193	0.88 (0.001 – 237.4)	0.97
PAT/B	0.1 (0.0004 – 14.8)	0.38	0.1 (0.0002 – 29.4)	0.45	0.002 (<0.0001 – 845.5)	0.35
EAT/B	0.013 (<0.0001 – 1692.2)	0.48	2.2 (<0.0001 – >2000)	0.96	0.001 (<0.0001 – 1028)	0.34
(SM + VAT)/B	0.14 (0.04 – 0.44)	**0.0005**	0.2 (0.06 – 0.69)	**0.01**	0.03 (<0.001 – 3.8)	0.16

Univariate Cox proportional hazard ratio (HR) analysis for the entire study cohort, male patients, and female patients. B,Bone; EAT, Epicardial Adipose Tissue; IMAT, Intramuscular Tissue; PAT, Pericardial Adipose Tissue; SAT, Subcutaneous Adipose Tissue; SM, Skeletal Muscle; TAT, Total Adipose Tissue; VAT, Visceral Adipose Tissue; 95%CI, 95% confidence interval.

Bold numbers indicate statistical significance and make significant factors easier to recognize.

**Table 3:** Uni- and multivariate survival analysis of pretreatment factors.

Groups		Median OS in months (95% CI)	Univariate analysis		Multivariate analysis	
			HR (95% CI)	P-value	HR (95% CI)	P-value
Gender	Female	4.8 (0.9 – 16.0)	1	**0.028**	1	0.6
	Male	18.5 (4.9 – 28.6)	0.38 (0.17 – 0.86)		0.73 (0.26 – 2.15)	
Age	>70 years	8.13 (1.1 – 25.6)	1	0.3	–	–
	≦ 70 years	9.6 (4.5 – 26.2)	0.68 (0.33 – 1.39)		–	
T-stage*	1	12.1 (1.13 –.)	1	0.12	–	–
	2	28.6 (2.3 –.)	0.44 (0.1 – 1.83)		–	
	3	9.6 (1.2 – 2.9)	1.19 (0.4 – 3.58)		–	
	4	8.1 (4.5 – 20.2)	1.62 (0.6 – 4.74)		–	
N-stage**	0	20.2 (1.13 –.)	1	0.8	–	**-**
	1	17.9 (5.2 – 28.6)	1.26 (0.37 – 4.3)		–	
	2	6.7 (2.3 – 21.9)	1.43 (0.59 – 3.47)		–	
	3	9.7 (0.9 –.)	1.73 (0.55 – 5.5)		–	
M-stage	0	6.5 (4.2 – 20.2)	1	0.1	–	–
	1	23.3 (2.7 –.)	0.51 (0.22 – 1.2)		–	
p16	Not positive	5.23 (3.4 – 18.5)	1	0.1	–	–
	positive	26.2 (6.9 -.)	0.49 (0.2 – 1.2)		–	
UICC	1	6.3 (0.23 –.)	1	0.68	–	–
	2	18.5 (1.1 – 37.8)	0.88 (0.21 – 3.7)		–	
	3	25.6 (1.1 –.)	0.49 (0.1 – 2.48)		–	
	4	6.5 (3.4 – 26.2)	0.99 (0.29 – 3.36)		–	
ECOG	0	28.9 (4.5 –.)	1	**0.028**	1	0.21
	1	8.1 (4.2 – 20.2)	2.1 (0.87 – 5.2)		2.5 (0.86 – 7.1)	
	2	3.4 (0.23 – 21.9)	4.5 (1.4 – 14.8)		3.0 (0.79 – 11.0)	
	3-4	1.5 (1.1 -.)	5.5 (1.5 – 20.1)		1.1 (0.25 – 5.0)	
BMI (kg/m^2)^)***	< 18.5	3.9 (1.1 – 9.6)	1	**0.02**	1	0.53
	≥ 18.5	17.6 (4.9 – 28.6)	0.34 (0.15 – 0.81)		0.73 (0.28 – 1.95)	
Albumin	≦ 3.4 g/dl	4.5 (2.3 – 9.6)	1	**0.0024**	1	**0.01**
	> 3.4g/dl	20.2 (7.9 –.)	0.32 (0.15 – 0.69)		0.31 (0.12 – 0.76)	
SM/B	≦ 1.79 (m)/1.56 (f)	4.5 (1.1 – 16.0)	1	**0.0008**	1	**0.004**
	> 1.79 (m)/1.56 (f)	21.9 (6.5 – 38.0)	0.26 (0.12 – 0.58)		0.25 (0.1 – 0.64)	
(SM + VAT)/B	≦ 2.0 (m)/1.74 (f)	5.2 (2.1 – 16)	1	**0.0035**	–	–
	> 2.0 (m)/1.74 (f)	28.6 (4.9 -.)	0.338 (0.16 – 0.72)		–	

Uni- and multivariate survival analyses of pretreatment factors. BMI, Body Mass Index; B, bone; ECOG, Eastern Cooperative Oncology Group; HR, Hazard Ratio) with 95% confidence interval, M, Metastases; N, Lymph Node; SM, Skeletal Muscle; T, Tumor; VAT, Visceral Adipose Tissue. * Tumor-stage (1–2 vs. 3-4) and **nodal-stage positive vs. negative were both not statistically significant in the Cox proportional hazard ratio analysis. ***No statistical difference on OS was seen for patients with normal (up to 24.9 kg/m^2^) and elevated (> 24.9 kg/m^2^) BMI.

Bold numbers indicate statistical significance and make significant factors easier to recognize.

Univariate analysis revealed that SM/B, (SM + VAT)/B, sex, albumin (albumin ≤ 3.4 g/dl vs. > 3.4 g/dl), ECOG performance score, and BMI (< 18.5 kg/m^2^ vs. ≥ 18.5 kg/m^2^) were statistically significant in univariate analysis (**Table 3**). Owing to the very high correlation between SM/B and (SM+VAT)/B (r^2^:0.73, p <0.0001), only SM/B was included in the multivariate analysis due to the lower hazard ratio (0.26 vs. 0.34). Multivariate analysis revealed that the SM/B ratio and albumin levels ≤ 3.4 g/dl vs. > 3.4 g/dl were statistically significantly independent, with a lower hazard ratio for the SM/B ratio compared to albumin (0.25 vs. 0.31).

**Figure 3:**
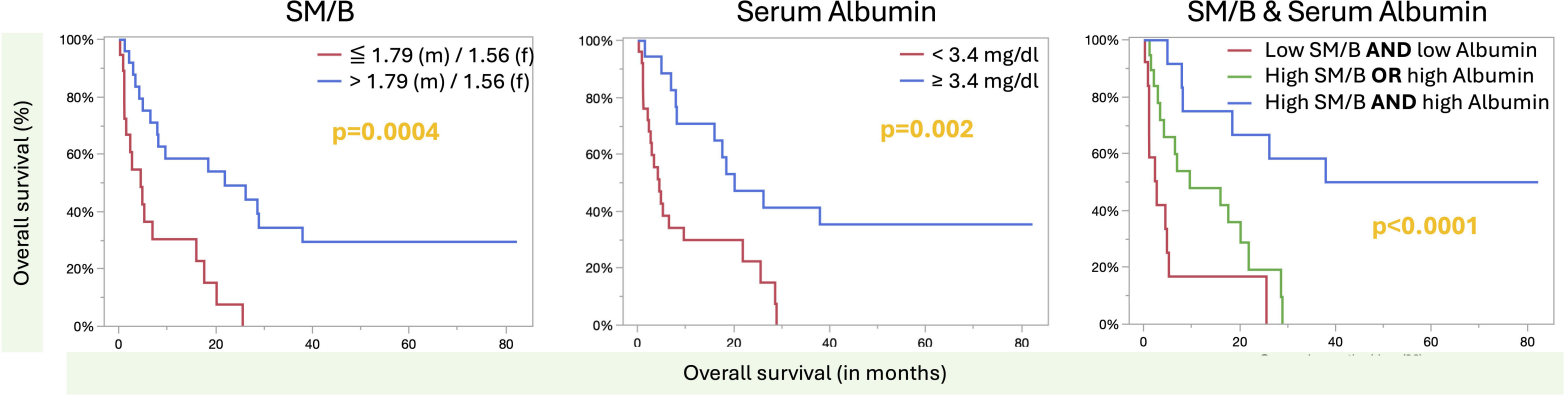
Overall survival according to skeletal muscle-to-bone ratio and serum albumin. The Kaplan-Meier analysis of SM/B (left), serum albumin (middle), and the scoring based on SM/B and serum albumin is presented here. P-values are derived from log-rank testing. The risk-based assessment of SM/B + serum albumin revealed statistically significant survival times across all groups, with a p-value of 0.03 or less.

To more accurately stratify the patients’ survival risk, a risk assessment was conducted using both significant factors from the multivariate analysis for scoring. Here, the longest survival was observed in patients with normal albumin levels and a higher SM/B ratio, with median survival time not reached (95% CI: 7.9 –. months). Patients with either a lower serum albumin level or a lower SM/B ratio survived for a median of 9.6 months (95% CI: 3.4 – 21.9), whereas patients with a lower serum albumin level and a lower SM/B ratio survived for a median of 2.7 months (95% CI: 0.87 – 5.2) (log-rank: p<0.0001). The overall survival curves for SM/B, albumin, and the combined scoring analysis are shown in **Figure 3**.

CT-based follow-up imaging of the thorax was available for 26 patients at 3 months. Here, a decrease in the SM/B ratio of ≥ 8% occurred in six (23.1%) patients, with a significantly shorter survival time of 6.7 (95% CI: 3.4 – 17.6) vs. 26.2 (95% CI: 7.9 – 38.0) months (log-rank: p = 0.032) and an increased hazard ratio of 3.7 (95% CI: 1.26 – 10.6; p = 0.017). Compared to the baseline ratios, there was no statistical difference between patients with a high SM/B decrease and those without, with 1.69 (95% CI: 1.42–1.95) versus 1.67 (95% CI: 1.52–1.81), p = 1.0. Of note, no statistical difference was observed in the gender comparison as well. Additionally, none of the patients with an ECOG score of 0 experienced a SM/B loss of more than 8%, whereas this was observed in 35.3% of patients with an ECOG score of 1 or higher.

### 3.3 Time to progression analysis

The median time to progression was 7.5 months (95% CI: 2.7 – 12.5) with 1-, 3-, 6 months and 1-, 2-, and 3-year progression-free rates of 87.8%, 56.1%, 51.2%, 34.2%, 19.5%, and 12.2%, respectively (**Figure 2B**). The evaluation of the SM/B ratio concerning disease control after three months revealed a significantly higher median SM/B ratio of 1.77 compared to 1.47 (p = 0.002) for patients with early tumor progression. In subgroup analysis, this difference was statistically significant in male participants (1.79 vs. 1.55; p = 0.035), but did not reach statistical significance in female participants (1.57 vs. 1.35; p = 0.2). Notably, the time to progression exhibited a significant correlation with the SM/B ratio (r² = 0.18; p = 0.006).

In **Table 4**, the significant parameters from the univariate analysis are presented, where albumin was the only independent variable associated with time to progression. Notwithstanding, the utilization of SM/B and albumin for further risk stratification, analogous to survival analysis, indicated that the most prolonged time to progression occurred in patients with normal albumin levels greater than 3.4 g/dL and a higher SM/B ratio, with a median TTP of 32.6 months (95% CI: 2.4 –.). Patients with either a high serum albumin level or a high SM/B ratio had a median TTP of 7.5 months (95% CI: 2.6 – 13.2), whereas patients with a low serum albumin level and a low SM/B ratio progressed after a median of 2.3 months (95% CI: 0.1 – 4.1) (log-rank: p=0.001). All groups were statistically significant (p ≤ 0.048). Notably, patients with a decrease in the SM/Ratio experienced a shorter time to progression of 2.98 months (95% CI: 4.1–32.6) compared to 12.0 months (95% CI: 4.1–32.6), though this difference was not statistically significant (p = 0.052).

**Table 4:** Uni- and multivariate time to progression analysis of pretreatment factors.

Groups		Median overall survival in months (95% CI)	Univariate analysis		Multivariate analysis	
			HR (95% CI)	P-value	HR (95% CI)	P-value
Gender	Female	2.7 (0.13 – 13.2)	1	**0.031**	1	0.4
	Male	11.5 (3.2 – 16.2)	0.38 (0.17 – 0.87)		0.6 (0.18 – 2.0)	
M-stage	0	3.2 (2.4 – 12.3)	1	**0.018**	1	0.16
	1	Not reached (2.8 –.)	0.35 (0.13 – 0.91)		0.46 (0.16 – 1.3)	
ECOG	0	11.9 (2.9 –.)	1	**0.038**	1	0.28
	1	7.5 (2.3 – 12.5)	1.7 (0.71 – 4.2)		2.6 (0.85 – 8.4)	
	2	2.8 (0.37 – 14.5)	2.7 (0.8 – 9.3)		1.66 (0.37 – 7.6)	
	3-4	1.5 (0.1 – 2.3)	11.5 (2.4 – 55.0)		4.1 (0.79 – 21.8)	
BMI (kg/m^2)^)	< 18.5	2.7 (0.1 – 16.2)	1	**0.02**	1	0.77
	≥ 18.5	10.2 (2.8 – 13.2)	0.34 (0.15 – 0.81)		0.85 (0.28 – 2.6)	
Albumin	≦ 3.4 g/dl	2.9 (2.3 – 10.2)	1	**0.013**	1	**0.03**
	> 3.4g/dl	13.2 (2.7 –.)	0.38 (0.17 – 0.83)		0.34 (0.13 – 0.92)	
SM/B	≦ 1.79 (m)/1.56 (f)	2.9 (0.4 – 11.6)	1	**0.014**	1	0.058
	> 1.79 (m)/1.56 (f)	11.5 (2.8 – 32.6)	0.38 (0.18 – 0.8)		0.37 (0.14 – 1.02)	
(SM + VAT)/B	≦ 2.0 (m)/1.74 (f)	4.1 (1.2 – 10.1)	1	**0.0462**	–	**-**
	> 2.0 (m)/1.74 (f)	12.2 (2.9 -.)	0.47 (0.22 – 0.99)		–	

Uni- and multivariate time-to-progression analyses of pretreatment factors. All factors presented in **Table 3** were investigated, and only significant factors of the univariate analysis are presented. BMI, Body Mass Index; B, Bone; ECOG, Eastern Cooperative Oncology Group; HR, Hazard Ratio; SM, Skeletal Muscle; VAT, Visceral Adipose Tissue.

Bold numbers indicate statistical significance and make significant factors easier to recognize.

### 3.4 Immune-related adverse events

Immune-related adverse events were observed in 14 patients (31.1%), with a median onset of 11.5 weeks (range: 2–60 weeks) after treatment initiation. Only one patient experienced a grade 3 adverse event, vasculitis and hepatitis, which was recorded after 4 weeks of treatment with Pembrolizumab. The irAE rate was significantly higher in patients treated with Nivolumab (53%) than in those treated with Pembrolizumab (20%) (p = 0.023). There were no significant differences between patients with and without irAE regarding body composition parameters in group- and sex-specific testing (p > 0.05) or albumin levels, sex, and ECOG performance status. Furthermore, survival rates were comparable, with a median of 8.0 months (95% CI: 2.7 -) for patients with irAEs compared to 9.6 months (95% CI: 4.2-21.9) for patients without irAEs (p = 0.42).

## 4 Discussion

The present study demonstrates that the implementation of automated methodologies for evaluating body composition, with particular emphasis on the skeletal muscle-to-bone (SM/B) ratio, affects overall survival and progression-free intervals in patients with HNSCC undergoing immunotherapy. This finding aligns with the results of a meta-analysis encompassing over 2,300 patients, which revealed a higher hazard ratio for sarcopenic patients undergoing surgery (HR: 2.5, 95% CI: 1.95 – 3.21) or radiotherapy (HR: 1.63, 95% CI: 1.4 – 1.9) (13). These findings are consistent with our study’s results, which reported an HR of 3.85 (95% CI: 1.7–8.1), suggesting that the prognostic significance of sarcopenia extends to the immunotherapy setting, with important clinical implications given the increasing use of checkpoint inhibitors in the treatment of HNSCC.

The link between sarcopenia and HNSCC is intricate and influenced by several factors. The underlying causes of cancer and non-cancer-related factors, including aging, metabolic disturbances, inflammation, and restricted food intake due to large tumors or posttherapeutic changes, make patients with HNSCC more susceptible to the development and worsening of sarcopenia and frailty than patients with other types of cancer (6, 18, 19). Furthermore, skeletal muscle is a key regulator of systemic metabolism and immune function. Sarcopenia is associated with increased levels of pro-inflammatory cytokines (e.g., IL-6, TNF-α, and IL-15), which can promote an immunosuppressive tumor microenvironment and impair the activation, recruitment, and function of cytotoxic T cells, as well as enhancing the recruitment and activation of myeloid-derived suppressor cells, which may potentially reduce the efficacy of immune checkpoint inhibitors (20–24). Furthermore, muscle wasting, which may be more often present in patients with lower muscle mass, also leads to metabolic disturbances, including increased lactate and altered amino acid availability, which can further suppress effector T cell activity and favor tumor progression (23, 25). These metabolic changes in the microenvironment can drive immune cell exhaustion and reduce the efficacy of immune checkpoint inhibitors.

In contrast, greater muscle mass may help establish a stronger immune environment with beneficial oncological outcomes (24). This observation may also provide a rationale for the association between higher SM/B ratios and superior survival and enhanced disease control rates. However, given that SM/B was not an independent factor for TTP in multivariate analysis, it is necessary to assume that further factors influence muscle mass and tumor control.

Skeletal muscle is unquestionably the most significant and extensively studied body composition parameter in the context of HNSCC patient outcomes. However, previous CT-based imaging studies have also examined the role of VAT in HNSCC. These studies have demonstrated that patients with higher VAT have a longer survival rate. In contrast, those with a significant decrease in VAT after three months of treatment with surgery and chemoradiation therapy have a shorter survival rate (26, 27). In contrast, the VAT/B ratio was not significant in this study cohort.

Furthermore, combining skeletal muscle and visceral adipose tissue did not improve outcome prediction compared with SM/B alone. This adverse finding may be explained by the fact that only small parts of the upper abdomen were included, and these parts may vary in volume, for example, due to differences in stomach filling status. Moreover, the aforementioned studies examined patients who underwent surgical procedures and chemoradiotherapy, excluding those who received immunotherapy, which might also exert an effect. In summary, VAT of the upper abdomen is not an additive prognostic factor in this analysis, either in isolation or in combination with skeletal muscle, for risk factor assessment of outcomes.

A weight reduction exceeding 2% within six weeks (35% of the study population) has been documented as a predictor of diminished survival in HNSCC patients undergoing treatment with immune checkpoint inhibitors (28). A comparable association was observed with the 8% decline in the SM/B ratio over three months, indicative of muscle wasting.

However, the selected cutoff may be constrained by several factors: Patients without follow-up imaging had a median overall survival of 2.3 months, compared with 17.3 months for patients with follow-up imaging (p = 0.077). Notwithstanding the lack of statistical significance, which may be attributed to the modest sample size, this discrepancy could introduce bias, potentially overlooking even more substantial and earlier muscle mass loss in patients not undergoing follow-up imaging. Furthermore, despite the Sørensen Dice score of 0.9553 for this model, indicating excellent segmentation accuracy in medical imaging (14), the possibility of measurement inaccuracies may result in higher variations, which could potentially reduce sensitivity and specificity. Overall, Further research is necessary to investigate the clinical implications of this measurement method and to determine effective countermeasures to ameliorate outcomes.

It is worth noting that previous studies vary in their methodologies for assessing body composition parameters, with the vast majority evaluating only a single slice, whereas this study evaluates the entire thoracic cavity. Additionally, extant studies primarily rely on abdominal imaging, with fewer studies employing cervical CT scans and even fewer employing thoracic CT scans (6, 12, 13). Given the observation that abdominal CT imaging for staging in HNSCC patients is performed less frequently than thoracic CT imaging, the extraction of opportunistic body composition parameters from thoracic scans can be performed more consistently without the need for additional abdominal imaging. It is noteworthy that the recent modification of the German guideline on oro- and hypopharyngeal carcinoma now includes abdominal CT at baseline staging, thereby increasing the availability of abdominal CT scans for future body composition analysis (29). Although CT imaging of the neck is more routinely employed, it is imperative to acknowledge the potential for bias arising from post-therapeutic alterations resulting from surgery and radiotherapy in body composition analysis. Moreover, distinguishing tumorous tissue from skeletal muscle can be challenging, and the presence of hardening artifacts from dental implants can, in some cases, substantially compromise the reliability of body composition analysis due to inferior delineation of target structures.

Overall, the application of automated body composition analysis of the thoracic cavity offers numerous benefits, including the accessibility of existing imaging data, seamless integration of automated sarcopenia assessment into the clinical workflow, avoidance of additional workload, and reduction of inter-observer variability. Additionally, the body composition assessment was performed solely using CT-based information, without the need to standardize for body height, as is usually necessary in conventional CT-based methods. Beyond its established application in various clinical cohorts, including HCC (30), pancreatic cancer (31), colorectal cancer (32) and the Health ABC study (33), internal normalization via bone volume is further supported by the strong biological link between muscle and bone volumes (34, 35). Notably, this correlation is superior to that of standard anthropometric metrics, such as body height and Body Mass Index (35) which are often used for normalization in previous studies. Due to inconsistent and unreliable reporting of body size and height within this study cohort, a direct comparison was not feasible. Overall, this approach further increases the utility of baseline scans by transforming them from purely descriptive assessments into tools with prognostic and predictive relevance. Nonetheless, further evaluation is necessary to validate or refine the cutoff values for body composition parameters and to determine the most effective methods for incorporating this analysis into a comprehensive treatment approach for patients, thereby ensuring optimal benefits from the analysis.

Consistent with other studies reporting similar findings in patients with HNSCC treated with immunotherapy, hypoalbuminemia is an independent predictor of overall survival and time to progression (36–38) This is because it is linked to inflammation, metabolic dysregulation, and disease severity, thereby affecting prognosis (39). The pharmacokinetics of the administered Pembrolizumab and Nivolumab are closely associated with the neonatal Fc-receptor pathway, which is also responsible for maintaining albumin levels in the body. Low serum albumin levels, indicating increased protein catabolism, also affect the breakdown of Pembrolizumab and Nivolumab, resulting in lower drug concentrations in the blood. Consequently, patients with hypoalbuminemia may experience reduced drug exposure, which can lead to less effective treatment and shorter survival times, as observed in the present study (40). Given the distinct underlying mechanisms of muscle mass and serum albumin in determining outcomes, as well as their potential for risk stratification based on SM/B and serum albumin in predicting overall survival and time to progression, combining these markers could help estimate potential treatment benefits for HNSCC patients undergoing immunotherapy in clinical routine, as demonstrated in this study.

This study has several limitations. First, the study was retrospective with a small sample size, and data collection was limited to a single center. Not all patients treated with immunotherapy had pretherapeutic CT scans available in the institutional imaging archive, which could have introduced a selection bias. Additionally, the quantification method used for body composition analysis differs substantially from that in previous studies, preventing meaningful comparisons or the transfer of cutoff values. Consequently, the sex-specific SM/B thresholds identified in this cohort require further validation and refinement. Given the limited number of female participants, the lack of statistical significance in this subgroup may reflect insufficient statistical power, posing a risk of Type II errors (false negatives) and warranting future studies with larger sample sizes.

## 5 Conclusion

This study demonstrates that automated body composition analysis, particularly the SM/B ratio, provides valuable prognostic information for patients with HNSCC undergoing immunotherapy. The integration of body composition metrics with conventional prognostic factors, such as albumin, facilitates enhanced risk stratification, potentially informing clinical decision-making and patient management strategies. The implementation of automated assessment methods enables routine evaluation of sarcopenia in clinical practice, with the potential to improve outcomes by identifying high-risk patients and implementing targeted interventions. Further research is needed to validate these findings and develop evidence-based interventions for sarcopenia in patients with HNSCC who are receiving immunotherapy.

## Data Availability

The raw data supporting the conclusions of this article will be made available by the authors, without undue reservation.
