# Supplementary material for: Muscle matters: automated CT-based body composition analysis predicts survival in patients with head and neck cancer treated with immunotherapy
Source: Front Oncol. 2026 Feb 24;16:1725892. doi: 10.3389/fonc.2026.1725892 (PMC12971423; doi:10.3389/fonc.2026.1725892)
Supplement: Supplementary file 1 [file Table1.docx]

Supplementary Table 1: Median values of body composition parameters

| **Body composition parameter** | **All patients (IQR)** | **Female patients (IQR)** | **Male patients (IQR)** | **p-value** |
| --- | --- | --- | --- | --- |
| SM/B | 1.65 (1.44–1.81) | 1.44 (1.32–1.53) | 1.69 (1.49–1.91) | **0.003** |
| TAT/B | 2.01 (1.54–2.77) | 2.79 (2.12–3.54) | 1.85 (1.29–2.58) | **0.022** |
| IMAT/B | 0.48 (0.34–0.68) | 0.64 (0.50–0.71) | 0.45 (0.32–0.64) | 0.105 |
| SAT/B | 1.12 (0.84–1.49) | 1.78 (1.31–2.63) | 1.01 (0.78–1.32) | **0.002** |
| VAT/B | 0.18 (0.12–0.28) | 0.15 (0.09–0.17) | 0.22 (0.13–0.35) | 0.074 |
| PAT/B | 0.13 (0.09–0.16) | 0.14 (0.07–0.15) | 0.13 (0.09–0.16) | 0.845 |
| EAT/B | 0.05 (0.03–0.07) | 0.07 (0.04–0.07) | 0.04 (0.03–0.07) | 0.327 |
| PAT/B | 0.13 (0.09–0.16) | 0.14 (0.07–0.15) | 0.13 (0.09–0.16) | 0.845 |
| SM + VAT/B | 1.84 (1.57–2.06) | 1.51 (1.43–1.75) | 1.93 (1.71–2.14) | **0.001** |

Median values of standardized body composition parameters and ratios for the entire study cohort, male patients, and female patients with interquartile ranges (IQR). Abbreviations: B (Bone), EAT (Epicardial Adipose Tissue), IMAT (Intramuscular Tissue), PAT (Pericardial Adipose Tissue), SAT (Subcutaneous Adipose Tissue), SM (Skeletal Muscle), TAT (Total Adipose Tissue), VAT (Visceral Adipose Tissue).

Supplementary Table 2: Area under the curve evaluation for body composition analysis for all patients and stratified for gender

| **Body composition Parameter** | **All patients** | | | **Male patients** | | | **Female patients** | | |
| --- | --- | --- | --- | --- | --- | --- | --- | --- | --- |
|  | AUC (95% CI) | p-value | cutoff | AUC (95% CI) | p-value | cutoff | AUC (95% CI) | p-value | cutoff |
| SM/B | 0.74 (0.59 – 0.86) | **0.0069** | >1.561 | 0.76 (0.59 – 0.89) | **0.001** | >1.794 | 0.63 (0.31 – 0.89) | 0.48 | >1.561 |
| TAT/B | 0.57 (0.42-0.72) | 0.21 | >1.54 | 0.62 (0.44 – 0.78) | 0.3 | >1.019 | 0.63 (0.31 – 0.89) | 0.45 | >3.63 |
| IMAT/B | 0.59 (0.43 – 0.73) | 0.2 | >0.631 | 0.59 (0.41 – 0.76) | 0.42 | >0.125 | 0.77 (0.43 – 0.96) | 0.09 | >0.632 |
| SAT/B | 0.59 (0.43 – 0.73) | 0.29 | >1.1 | 0.65 (0.47 – 0.81) | 0.17 | >0.96 | 0.63 (0.31 – 0.89) | 0.46 | >1.0952 |
| VAT/B | 0.6 (0.44 – 0.74) | 0.2 | >0.1624 | 0.57 (0.39 – 0.74) | 0.55 | >0.083 | 0.57 (0.35 – 0.85) | 0.72 | >0.179 |
| PAT/B | 0.65 (0.49 – 0.78) | 0.09 | >0.086 | 0.6 (0.42 – 0.76) | 0.4 | >0.073 | 0.73 (0.4 – 0.94) | 0.17 | >0.095 |
| EAT/B | 0.57 (0.41 – 0.72) | 0.39 | >0.024 | 0.63 (0.44 – 0.78) | 0.28 | >0.029 | 0.53 (0.23 – 0.82) | 0.86 | >0.061 |
| (SM + VAT)/B | 0.76 (0.61 – 0.82) | **0.0032** | >2.0 | 0.79 (0.6 – 0.91) | **<0.001** | >2.0 | 0.7 (0.37 – 0.93) | 0.238 | >1.741 |

Area under the curve (AUC) analysis for the entire study cohort, male patients, and female patients. Cutoff points were determined using Youden’s index based on a survival of three months. Abbreviations: B (Bone), EAT (Epicardial Adipose Tissue), IMAT (Intramuscular Tissue), PAT (Pericardial Adipose Tissue), SAT (Subcutaneous Adipose Tissue), SM (Skeletal Muscle), TAT (Total Adipose Tissue), VAT (Visceral Adipose Tissue).
